# Population Genetic Diversity and Structure of a Naturally Isolated Plant Species, *Rhodiola dumulosa* (Crassulaceae)

**DOI:** 10.1371/journal.pone.0024497

**Published:** 2011-09-01

**Authors:** Yan Hou, Anru Lou

**Affiliations:** State Key Laboratory of Earth Surface Processes and Resource Ecology, College of Life Sciences, Beijing Normal University, Beijing, China; Montreal Botanical Garden, Canada

## Abstract

**Aims:**

*Rhodiola dumulosa* (Crassulaceae) is a perennial diploid species found in high-montane areas. It is distributed in fragmented populations across northern, central and northwestern China. In this study, we aimed to (i) measure the genetic diversity of this species and that of its populations; (ii) describe the genetic structure of these populations across the entire distribution range in China; and (iii) evaluate the extent of gene flow among the naturally fragmented populations.

**Methods:**

Samples from 1089 individuals within 35 populations of *R. dumulosa* were collected, covering as much of the entire distribution range of this species within China as possible. Population genetic diversity and structure were analyzed using AFLP molecular markers. Gene flow among populations was estimated according to the level of population differentiation.

**Important Findings:**

The total genetic diversity of *R. dumulosa* was high but decreased with increasing altitude. Population-structure analysis indicated that the most closely related populations were geographically restricted and occurred in close proximity to each other. A significant isolation-by-distance pattern, caused by the naturally fragmented population distribution, was observed. At least two distinct gene pools were found in the 35 sampled populations, one composed of populations in northern China and the other composed of populations in central and northwestern China. The calculation of Nei's gene diversity index revealed that the genetic diversity in the northern China pool (0.1972) was lower than that in the central and northwestern China pool (0.2216). The populations were significantly isolated, and gene flow was restricted throughout the entire distribution. However, gene flow among populations on the same mountain appears to be unrestricted, as indicated by the weak genetic isolation among these populations.

## Introduction

Habitat fragmentation is a significant threat to the maintenance of biodiversity in many terrestrial ecosystems [1]. Many studies have investigated the effects of fragmentation on the genetic diversity and population structure of plant species [2]–[4]. In general, fragmentation is expected to reduce genetic diversity and to increase interpopulation genetic divergence by restricting gene flow among fragmented populations, increasing inbreeding and increasing random genetic drift within populations [5]. However, habitat fragmentation does not always lead to reduced genetic variation [6]–[8]. In some cases, the genetic diversity of a fragmented population can be higher than that of a continuously distributed population [9]. This is because the effects of habitat fragmentation on genetic diversity and population structure can be affected by other factors, such as population size, gene flow and the time scale of fragmentation [10].

Fragmented distributions of plant populations are caused not only by human activity but also by natural factors, such as long-term, large-scale climate oscillations, topographical changes, the isolation of suitable habitats, or other ecological changes. Studies of the genetic diversity of naturally fragmented populations may not only reveal the ecological consequences of population fragmentation over long periods of time but also provide a frame of reference for predicting the consequences of habitat fragmentation by human activities [6]. Genetic analyses of naturally fragmented populations has been conducted in several studies [6], [11]–[14].

*Rhodiola dumulosa* (Crassulaceae) is a perennial plant species that is found in northern, central and northwestern China. *R. dumulosa* is suggested to be a diploid plant [15]. It uses a mixed-mating system, i.e., it is capable of self-fertilization but most often undergoes outbreeding via pollinators [16], [17]. This species lives exclusively between rocks on mountains at elevations of 1600 m to 4100 m. Wild populations of this species are uniquely adapted to scarce and highly fragmented rocky habitats. Its habituation to these ‘ecological islands’ has produced a naturally fragmented distribution, which may lead to the long-term genetic isolation of its populations. Most populations in our field investigation were of moderate size (around 50 to 80 individuals each population). However, we also found a few large populations, with more than 150 individuals, and very small populations, with fewer than 10 individuals.

In this study, we used AFLP analysis to assess the genetic diversity, differentiation and structure of isolated populations of *R. dumulosa* from different mountains across its entire distribution in China. We then used these results to address the following questions: (1) How much genetic diversity is maintained in these naturally fragmented populations of *R. dumulosa*? (2) How is the genetic diversity spatially structured across the entire distribution range of this species in China? (3) Is there any gene flow among these naturally fragmented populations?

## Results

Four AFLP primer combinations were used to analyze 1,089 individual genotypes, resulting in 225 markers, 221 (98.22%) of which were polymorphic.

### Population genetic structure

**Figure 1:**
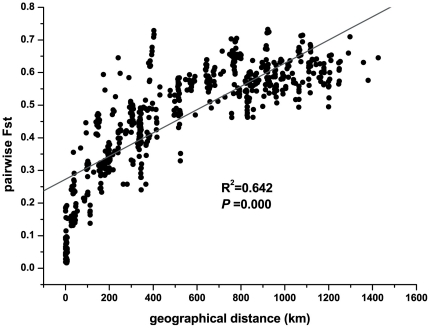
The correlation between pairwise Fst and pairwise geographic distance among populations of *Rhodiola dumulosa.*

The Mantel test (Fig. 1) revealed a strong and significant positive relationship between geographical and genetic distances (*r* = 0.801; *P*<0.01) across the whole sampled region, indicating significant isolation-by-distance. We also conducted separate Mantel tests on the Donglingshan and Heyeping populations, in which populations were collected according to altitude, to determine whether isolation-by-distance also occurred on a smaller geographical scale. These results did not indicate any significant correlation between genetic differentiation and geographical distance within these populations (Donglingshan: *r* = 0.246; *P* = 0.186; Heyeping: *r* = 0.0852; *P* = 0.38).

**Figure 2:**
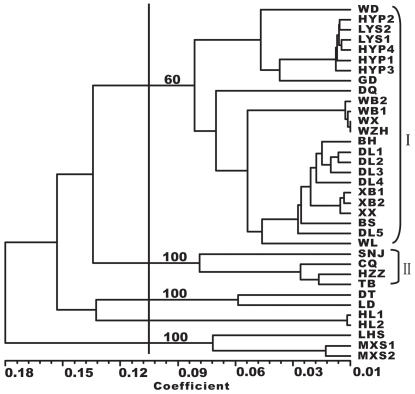
UPGMA tree of 35 *Rhodiola dumulosa* populations (numbers indicate bootstrap support values).

**Figure 3:**
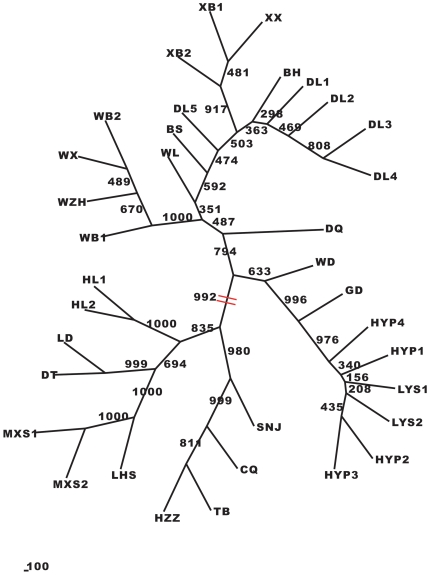
NJ tree of 35 *Rhodiola dumulosa* populations (numbers indicate bootstrap support values).

Hierarchical cluster analysis (UPGMA) showed that populations sampled from northern China and central China were grouped separately according to their geographical distribution (Fig. 2). Cluster I, with a bootstrap value of 60%, contained all populations in northern China, including 6 populations in Beijing, 5 populations in Hebei province, 12 populations in Shanxi province and 1 population in the Inner Mongolia autonomous region. Cluster II, with a bootstrap value of 100%, contained all populations in central China, including 1 population in Hubei province and 3 populations in Shaanxi province. The remaining northwestern populations could not be grouped in a single cluster, but the most closely related populations were geographically restricted and occurred in close proximity to each other. The NJ dendrogram revealed an obvious split between the populations in northern China and the other populations, with a bootstrap value of 99.2% (Fig. 3).

**Figure 4:**
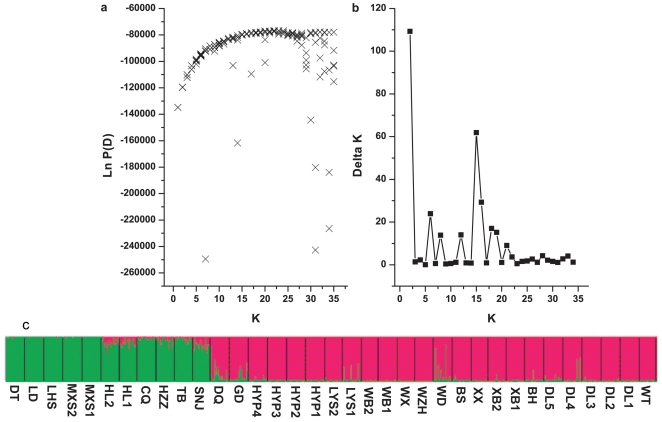
STRUCTURE analysis of *Rhodiola dumulosa* populations. Based on AFLP data (a: the relationship between K and LnP(D); b: the relationship between K and ΔK; c: the grouping when K = 2).

**Figure 5:**
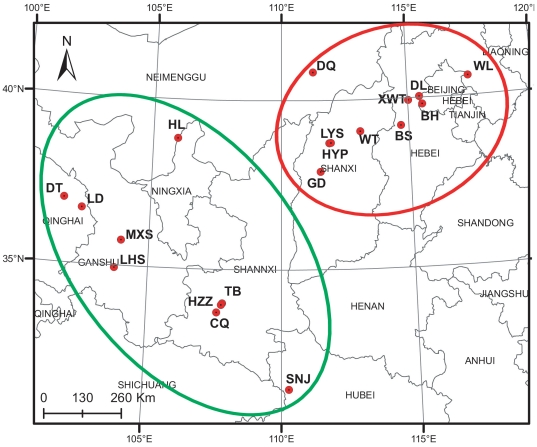
Distribution of the field sampling sites in China (the color of circles corresponds to two clusters resulted from STRUCTURE).

To further test this population structure, a model-based clustering method was implemented in the program STRUCTURE [18]–[20]. Without prior information about the populations and under an admixed model, STRUCTURE calculated that the estimate of the likelihood of the data (LnP(D)) was greatest when K = 2. For K>2, LnP(D) increased slightly but more or less plateaued (Fig. 4a), i.e., ΔK reached its maximum at K = 2 (Fig. 4b), suggesting that all populations fell into one of the two clusters. These two genetically distinct clusters primarily correspond to the geographic distribution of these populations (Fig. 5), and the percent representation of each cluster in each sampled population was high (Table S1). The red cluster covered all populations in northern China, and the remaining populations were grouped in the green cluster(Fig. 4c). This result is identical to the splitting in the NJ tree. Furthermore, UPGMA Cluster I is identical to the red cluster, indicating that the grouping of northern populations is well supported. Overall, the cluster analysis strongly suggested that the 35 sampled populations can be divided into two clusters, one composed of populations in northern China (NC) and the other composed of populations in central and northwestern China (CNWC).

Analysis of Molecular Variance (AMOVA) based on the two genetic clusters indicated that majority of genetic variation (43.49%) occurred within populations, while the variation between the two clusters was 23.68% (Table 1). AMOVA based on three major distribution regions gave a very similar result (42.59% variation within populations, 29.39% variation among the three regions). Moreover, AMOVA that treated the central population as one group calculated much higher genetic variation within populations (65.44%) than that among populations (34.56%), which may partly explain why the northern populations always clustered together as a group. The central populations also exhibited a higher genetic variation within populations (58.87% variation within the population and 41.13% variation among the three regions), while the northwestern populations had a slightly higher variation among populations than within populations (52.41% and 47.59%, respectively). When the central and northwestern populations were pooled together, AMOVA calculated somewhat higher genetic variation among populations (56.90%). We also treated the 35 populations as one group and compared the variation within and among populations. The Fst value was 0.49922 (*P*<0.001) with 50.08% within populations and 49.92% among populations, indicating that the total genetic variation was almost equally divided between intrapopulation and interpopulation variations. This approximately equal partitioning could have been the result of significant differentiation among the populations in northwestern China and frequent gene flow among some populations in northern China; we sampled more closely distributed populations in northern China than in northwestern China. Therefore, we randomly chose one population from each locality (resulting in the exclusion of populations DL2, DL3, DL4, DL5, XB1, XX, WD, WB1, WB2, WX, LYS2, HYP2, HYP3, HYP4, HL2, and MXS2) and repeated the analyses (Table 1). After excluding these populations, the Fst became 0.54252 (*P*<0.001) with 54.25% of variation occurring among populations, only slightly higher than the variation within populations. Therefore, we conclude that the differentiation among groups was significant, but the genetic variation within populations was maintained.

**Table 1:** Results of AMOVA for *R. dumulosa* individuals based on 225 AFLP markers.

Group	Partitioning	d.f.	Sum of squares	Variance components	Percentage of variation	F-statistics
two genetic clusters (NC & CNWC)	Among groups	1	3686.335	7.15780	23.68	Fct = 0.23679*
	Among populations within groups	33	10623.007	9.92465	32.83	Fsc = 0.43019*
	Within populations	1054	13855.386	13.14553	43.49	Fst = 0.56512*
	Total	1088	28164.728	30.22798		
three geographical regions (northern, central, northwestern)	Among groups	2	5277.625	9.07061	29.39	Fct = 0.29388*
	Among populations within groups	32	9031.718	8.64878	28.02	Fsc = 0.39684*
	Within populations	1054	13855.386	13.14553	42.59	Fst = 0.57409*
	Total	1088	28164.728	30.86492		
Northern populations	Among populations	23	5408.968	7.13192	34.56	Fst = 0.34560*
	Within populations	722	9750.255	13.50451	65.44	
	Total	745	15159.224	20.63643		
Central populations	Among populations	3	643.064	6.66237	41.13	Fst = 0.41129*
	Within populations	119	1134.839	9.53646	58.87	
	Total	122	1777.902	16.19883		
Northwestern populations	Among populations	6	2979.686	15.35977	52.41	Fst = 0.52414*
	Within populations	213	2970.292	13.94503	47.59	
	Total	219	5949.977	29.30480		
Central and Northwestern populations	Among populations	10	5214.039	16.32634	56.90	Fst = 0.56904*
	Within populations	332	4105.130	12.36485	43.10	
	Total	342	9319.169	28.69119		
19 populations from each locality	Among populations	18	8801.864	15.25624	54.25	Fst = 0.54252*
	Within populations	574	7384.328	12.86468	45.75	
	Total	592	16186.192	28.12092		

### Population genetic diversity

**Figure 6:**
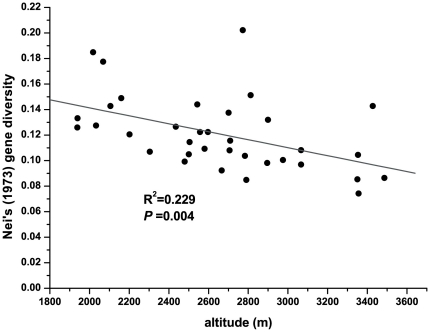
Scatter diagram of Nei's gene diversity and altitude.

The genetic diversity of each population was calculated based on the genotypes present (Table S2). The expected heterozygosity (or Nei's gene diversity, Hj) varied from 0.09690 in population MXS1 to 0.22679 in population WD. Similar numbers were calculated for the Shannon diversity index, which varied from 0.1171 in population MXS1 to 0.3174 in population WD. The total diversity of the species (Ht) was 0.2473. When the populations were divided into two clusters based on the STRUCTURE analyses, the Nei's gene diversity index was lower in the northern region of China (0.1972) than in the central and northwestern areas (0.2216). There was a significant negative correlation between population genetic diversity and altitude (*r* = −0.478, *P* = 0.004) across the entire range of *R. dumulosa*. A regression analysis of Nei's genetic diversity and altitude using SPSS 15.0 revealed that population diversity decreases with increasing altitude (Fig. 6).

### Gene flow

The genetic differentiation among 35 populations of *R. dumulosa* was high and significant (Fst = 0.3942, *P*<0.001; Gst = 0.4675). The estimated gene flow, Nm (Nm = 0.5(1− Gst)/Gst), was 0.5695. The results of this analysis reveal that the genetic differentiation among populations throughout the entire distribution area is significant and that gene flow is restricted. Furthermore, the populations in northern China always congregated together as a cluster (UPGMA tree, NJ tree and STRUCTURE analysis results). The genetic differentiation, Gst, among all northern populations was 0.3155, and the Nm was 1.0847. Thus, the gene flow among populations in northern China was much higher than that across the entire range.

On some mountains, more than one population was sampled. For instance, there were five populations in Donglingshan and four populations in Heyeping (Table 2). To investigate the gene flow among populations in these smaller geographical regions, the Gst and Nm of Donglingshan (DL1, DL2, DL3, DL4, DL5) and Heyeping (HYP1, HYP2, HYP3, HYP4) populations were calculated. The Gst of the five populations in Donglingshan was 0.1125, and the Nm was 3.9438. Meanwhile, the Gst of the four populations in Heyeping was 0.0728, and the Nm was 6.6390. These results indicated weak genetic differentiation and frequent gene flow within these small geographical regions. Consequently, to avoid the over-estimation of gene flow caused by frequent gene flow among populations on one mountain, we calculated the Gst and Nm of 19 populations with 593 individuals from only one population from each locality (as we did for AMOVA). These results revealed that the gene flow in small regions had a clear influence (Gst19 = 0.5437, Nm19 = 0.4197) on the gene flow in the entire region (Gst = 0.4675, Nm = 0.5695).

**Table 2:** Localities and sizes of the 35 *R. dumulosa* populations sampled in this study.

Sampling Locality	Population ID	Longitude (E)	Latitude (N)	Altitude (m)	Population size
Wulingshan, Hebei province	WL	117.461°	40.577°	1939	29
Donglingshan (1), Beijing	DL1	115.454°	40.031°	2303	32
Donglingshan (2), Beijing	DL2	115.462°	40.031°	2202	32
Donglingshan (3), Beijing	DL3	115.471°	40.034°	2106	32
Donglingshan (4), Beijing	DL4	115.475°	40.033°	2018	31
Donglingshan (5), Beijing	DL5	115.451°	40.029°	2160	32
Baihuashan, Beijing	BH	115.582°	39.813°	2033	32
Xiaowutaishan (beitai 1), Hebei	XB1	115.061°	39.945°	2500	31
Xiaowutaishan (beitai 2), Hebei	XB2	115.047°	39.943°	2706	30
Xiaowutaishan (xitai), Hebei	XX	114.968°	39.912°	2480	29
Baishishan, Hebei	BS	114.698°	39.208°	1940	32
Wutaishan (dongtai), Shanxi	WD	111.662°	39.041°	2773	32
Wutaishan (zhongtai), Shanxi	WZH	113.531°	39.047°	2895	30
Wutaishan (xitai), Shanxi	WX	113.492°	39.037°	2783	29
Wutaishan (beitai 1), Shanxi	WB1	113.568°	39.080°	3066	32
Wutaishan (beitai 2), Shanxi	WB2	113.568°	39.080°	3066	28
Luyashan (1), Shanxi	LYS1	111.926°	38.750°	2701	32
Luyashan (2), Shanxi	LYS2	111.926°	38.744°	2580	30
Heyeping (1), Shanxi	HYP1	111.837°	38.722°	2709	32
Heyeping (2), Shanxi	HYP2	111.862°	38.729°	2596	32
Heyeping (3), Shanxi	HYP3	111.874°	38.729°	2504	32
Heyeping (4), Shanxi	HYP4	111.885°	38.730°	2435	32
Guandishan, Shanxi	GD	111.518°	37.895°	2557	32
Daqingshan, Inner Mongolia	DQ	111.259°	40.838°	2068	31
Shennongjia, Hubei	SNJ	110.258°	31.446°	2974	30
Taibaishan, Shaanxi	TB	107.806°	33.997°	3486	30
Houzhenzi, Shaanxi	HZZ	107.767°	33.950°	3350	31
Changqing, Shaanxi	CQ	107.600°	33.717°	2790	32
Helanshan (west), Ningxia	HL1	105.944°	38.838°	3428	30
Helanshan (north), Ningxia	HL2	105.944°	38.846°	2900	30
Maxianshan (west), Gansu	MXS1	103.950°	35.750°	3356	32
Maxianshan (east), Gansu	MXS2	103.950°	35.750°	3353	32
Lianhuashan, Gansu	LHS	103.750°	34.933°	2812	32
Ledu, Qinghai	LD	102.389°	36.655°	2543	32
Datong, Qinghai	DT	101.691°	36.930°	2666	32

## Discussion

### Population diversity and its relationship to geographical distance and altitude

Our analysis of AFLP molecular markers indicated that *R. dumulosa* has maintained a high overall genetic diversity (Ht = 0.2473), similar to that of the two other species of *Rhodiola* but much higher than that of other perennial plants (Table 3). Thus, the expectation that genetic variability would decline due to population fragmentation was not supported in this case. This result might be observed because *R. dumulosa* still occurs in medium or large population sizes (40 to over 150 individuals) in some localities. Moreover, the gene flow among populations on the same mountain was high (Gst and Nm for Donglingshan and Heyeping populations). Another explanation could be that this high genetic diversity is a reflection of high historic genetic variability, which is quite common in long-lived perennial plant species [21]–[22].

**Table 3:** Comparison of genetic diversity of *R. dumulosa* with values from other *Rhodiola* species and selected other plant species.

Species	Genetic diversity	Marker	References conclusion
*Rhodiola species*			
*Rhodiola dumulosa*	PPL = 98.22%	AFLP	Present study
	Ht = 0.2473		
	I = 0.3625		
*Rhodiola rosea*	PPL = 95.3%	AFLP	high total genetic diversity [53]
	Ht = 0.266		
	I = 0.420		
*Rhodiola fastigiata*	PPL = 96.61%	AFLP	high total genetic diversity [54]
	Ht = 0.3329		
	I = 0.4893		
*Other plants*			
*Incarvillea younghusbandii*	Ht = 0.063	AFLP	low genetic diversity [55]
	I = 0.096		
*Cedrela odorata*	PPL = 98.8%	AFLP	high total genetic diversity [56]
	Ht = 0.22		

Analyses of the correlation between population genetic diversity and geographic altitude showed a weak but significant negative correlation. The lower genetic diversity of populations at higher altitudes might be the result of the smaller populations of pollinators in these areas; *R. dumulosa* is thought to breed by facultative xenogamy, which requires pollinators for outbreeding [16]. However, pollination biology studies of *R. dumulosa* concluded that populations in open fields, such as those near peaks, received more frequent pollinator visits than those at lower altitudes [17]. Therefore, we suggest that past climatic oscillations, which may have caused the local extinction and recolonization of populations at high altitudes, could have driven this pattern. The impacts of past climatic oscillations on species ranges and genetic structure have been addressed in several previous phylogeographic studies [23]–[25]. Here, we hypothesize that the extremely low temperatures experienced during glaciation resulted in the extirpation of *R. dumulosa* populations in high-altitude montane regions, whereas the populations in lower altitude areas with more favorable climate conditions survived. During interglacial periods, some offspring of the surviving low altitude populations recolonized the higher altitude. Thus, populations in higher altitude sites, derived from only a few individuals from lower altitude populations, would exhibit less genetic variation. Greater genetic diversity at in situ survival areas than at recolonized areas was also found in a number of phylogeographic studies, especially in the Alps [26]–[28]. For example, the genetic variation of a widespread alpine herb, Biscutella laevigata (Brassicaceae), reflected the influence of past climate changes on the species range as a gradient of genetic diversity along recolonization pathways [27], [29]. The study of these populations also revealed that the peripheral Alps are occupied by populations with significant haplotypic variation, whereas recently glaciated areas at high altitudes in the central Alps typically contain expanding populations with a single, fixed haplotype [29]. A third potential reason for the correlation between higher altitude and lower genetic diversity pattern is simply related to population size. It is widely accepted that genetic drift can have significant effects on small populations [30]. However, we did not observe an obvious gradient of population size according to altitude, and in some sampling areas, the populations on mountain peaks are larger because the mountain has a flat top (e.g., Wutaishan).

### Population structure and gene flow among naturally isolated populations

A strong correlation between genetic and geographical distances (Mantel test: r = 0.801; P<0.01) revealed a pattern of isolation-by-distance across the distribution range of *R. dumulosa* in China. This pattern suggested that the dispersal of this species might be constrained by distance such that gene flow is most likely to occur between neighboring populations [31]–[32]. As a result, more closely situated populations tend to be more genetically similar to one another [33]. The population genetic structure analyses (UPGMA tree, NJ tree and STRUCTURE) showed that populations tend to cluster in the same group when they are geographically restricted and occur in close proximity to one another. The congruence between the geographical distribution of populations and their genetic relationships is generally interpreted as sign of a longstanding pattern of highly restricted gene flow [34]. Our AFLP analysis revealed significant genetic differentiation and restricted gene flow among all sampled populations throughout the distribution range, which is naturally fragmented (Gst = 0.4675, Nm = 0.5695). This finding may be the result of the “ecological island” distribution pattern of *R. dumulosa* across its range in China. However, we also found that the gene flow among populations on the same mountain is relatively unrestricted, and the genetic differentiation among these populations is weak. For example, the Gst and Nm of the five populations in Donglingshan were 0.1125 and 3.9438, respectively, while the Gst and Nm of the four populations in Heyeping were 0.0728 and 6.6390. Furthermore, Mantel tests conducted on these two groups of populations revealed no significant correlations between genetic and geographical distances. Thus, our AFLP data suggest that although an isolation-by-distance pattern may be detected across the whole range of *R. dumulosa*, the gene flow and the relationship between geographical and genetic distances have different patterns at different spatial scales. Similarly distinct patterns at different spatial scales were also found for some other plant species [35].

The results of our hierarchical and model-based cluster analyses of AFLP data strongly suggested that the 35 sampled populations of *R. dumulosa* could be split into two clusters, one in northern China (NC) and the other in central and northwestern China (CNWC). In particular, the populations collected from northern China always clustered together regardless of the approach used for genetic structure analysis. AMOVA within the northern populations also clearly indicated that the genetic variation within populations was higher than that among populations (65.44% and 34.56%, respectively). Thus, we treated the northern populations as a reasonable cluster. However, when the populations from central and northwestern China were pooled together, AMOVA indicated that there was greater genetic variation among populations than within populations (56.90% and 43.10%), and the variation between NC and CNWC clusters was only 23.68%. Thus, the central and northwestern populations may not form a good cluster. More data may help resolve the genetic relationship between populations in these two areas. Moreover, when AMOVA was conducted based on different grouping approaches, we always found high genetic variation within populations (at least above 40%, Table 1). This finding could be due to some gene flow among populations, especially at small distribution scales, as discussed above. Alternatively, this result could reflect the preservation of ancient genetic diversity within populations as we also found significant genetic differentiation among populations or groups (Table 1). However, additional data, such as detailed gene sequence data, will be required to further dissect the evolutionary history of *R. dumulosa*.

In conclusion, the total genetic diversity of *R. dumulosa* was high, and population diversity decreased with increasing altitude. The geographical distribution of populations and their genetic relationships were consistent and most likely due to the natural geographic fragmentation of this species. A significant isolation-by-distance pattern was found across the entire distribution range in China, and significant genetic differentiation and restricted gene flow were observed among populations. However, restricted gene flow and a correlation between genetic and geographical distance were not appreciated at smaller spatial scales.

## Materials and Methods

### Population sampling and DNA extraction

Thirty-five populations of *R. dumulosa*, including 1089 individuals (28–32 individuals per population), were sampled from 19 localities across the entire distribution of this species in 2005, 2006 and 2007. The sampling area ranged from the northernmost population “DQ” in the Inner Mongolia Autonomous Region (40.838°N) to the southernmost population “SNJ” in Hubei province (31.446°N) and from the easternmost population “WL” in Hebei province (117.461°E) to the westernmost population “DT” in Qinghai province (101.691°E) (Fig. 5, Table 2). All sampled individuals were separated by at least five meters.

Leaf material was stored in zip-lock plastic bags with silica gel until DNA extraction. Sample vouchers were deposited in the collection at Beijing Normal University. Genomic DNA was extracted from silica gel-dried leaf material using a plant DNA extraction kit (Tiangen, Beijing, China) according to the manufacturer's protocol, with some modifications.

### AFLP fingerprinting

AFLP fingerprinting was performed according to the original protocol presented by Vos *et al.* [36], with minor modifications. Four pairs of primers (combinations of FAM-labeled *Eco*RI-ATG, *Mse*I-CTG, *Mse*I-CAA, *Mse*I-CAC and *Mse*I-CAG) were used for selective amplification. Selective amplification products were separated using an ABI 3100 sequencer (Applied Biosystems) with a GeneScan ROX 500 internal size standard. Electropherograms were then analyzed using GENEMAPPER 3.7 software (Applied Biosystems). To create a binary matrix, amplified fragments of 80–500 base pairs were scored visually as having present (1), absent (0), or ambiguous (?) peaks in the output traces. Only distinct peaks were scored as present, and the manual scoring procedure was repeated twice on separate occasions to reduce scoring errors.

### Genetic data analysis

The unweighted pair group method with averages (UPGMA) clustering analysis, derived from the Nei's minimum distance matrix [37], [38] as calculated in TFPGA v1.3 [39], was conducted using the SAHN module in NTSYS v2.10 [40]. One thousand bootstrapped replicate matrices of pairwise Fst among populations were calculated in AFLP-SURV. The results were used as inputs for computing Neighbor-Joining (NJ) dendrograms, using the NEIGHBOR module in the PHYLIP v3.68 [41] software package. An extended majority-rule consensus tree was produced using CONSENSE, a module for STRUCTURE v2.2 [20], adapted for dominant markers and used to assign an individual's probability of belonging to a homogeneous cluster (K populations) without prior population information. The correlated allele frequencies and admixed model were applied with a burn-in period of 100,000 and 1,000,000 MCMC replicates after burn-in. The range of clusters (K) was predefined from 1 to 35. From K = 1 to K = 10, ten runs were performed, whereas for K>10, five runs were performed. The Pr (X|K) (or “LnP(D)”) can be used as an indication of the most likely number of groups, and it usually plateaus or increases slightly after the “right K” is reached [42]. Therefore, the height of the modal value of the ΔK distribution was calculated to detect the true K [42] using Structure 2(1).2-sum [18]. The similarity coefficient of pair-wise runs for each value of K was also calculated in Structure 2(1).2-sum to control the stability of the results [43]. Permutations of the most likely results among different runs for each K were conducted in CLUMPP [44]. DISTRUCT [45] was used to visualize the STRUCTURE results. The partitioning of variation at different levels was calculated by Analysis of Molecular Variance (AMOVA) in ARLEQUIN v3.01 [46] using 1,000 permutations. The 35 populations were then grouped according to the clusters indicated by the UPGMA tree, NJ dendrograms and STRUCTURE analysis results, respectively. The correlation between the genetic (Fst) and geographic population pairwise distance matrices was evaluated using a Mantel test with 9999 permutations in ARLEQUIN v3.01, and the scatter plot was constructed in SPSS v15.0. The correlation between Nei's genetic diversity and altitude was calculated using SPSS v15.0. Genetic diversity and differentiation statistics were calculated using AFLP-SURV v1.0 [47], [48]. This program estimates allele frequencies at each marker locus in each population, assuming that the markers are dominant and that there are two alleles per locus (the presence of a band is considered dominant and its absence is considered recessive). A Bayesian method with a non-uniform prior distribution of allele frequencies [49] was used to estimate the allelic frequencies, which assumed Hardy-Weinberg equilibrium. These allele frequencies were then used to analyze the genetic diversity within and between samples according to the method described by Lynch and Milligan [50]. The proportions of polymorphic loci at the 5% level (PPL), as well as the expected heterozygosity (or Nei's gene diversity, Hj), standard errors (S.E.(Hj)) and total variance (Var(Hj)) were computed for each population. Wright's fixation index, Fst [51], was computed using the Lynch and Milligan [50] method and tested with a permutation procedure of 1000 replicates. POPGENE v1.32 [52] was also used to conduct descriptive analysis for AFLP markers. Nei's gene diversity (H, analogous to Hj), the Shannon diversity index, population differentiation (Gst) and the estimate of gene flow, Nm (Nm = 0.5(1 – Gst)/Gst) were also calculated.

## Supporting Information

Table S1 **Membership of each pre-defined population in each of the two clusters generated by CLUMPP based on the STRUCTURE v2.2 analysis (K = 2).**
(DOC)

Table S2 **Population genetic diversity of** ***R. dumulosa.*** (n: population size; PPL: proportion of polymorphic loci at the 5% level; Hj: expected heterozygosity or Nei's gene diversity; S.E.: standard error; Var: variance; H: Nei's gene diversity; I: Shannon diversity index; NC: northern China; CNWC: central and northwestern China).
(DOC)
