# Supplementary material for: Population Genetic Diversity and Structure of a Naturally Isolated Plant Species, Rhodiola dumulosa (Crassulaceae)
Source: PLoS One. 2011 Sep 1;6(9):e24497. doi: 10.1371/journal.pone.0024497 (PMC3164725; doi:10.1371/journal.pone.0024497)
Supplement: Table S1 — Membership of each pre-defined population in each of the two clusters generated by CLUMPP based on the STRUCTURE v2.2 analysis (K = 2). (DOC) [file pone.0024497.s001.doc]

Table S1: Membership of each pre-defined population in each of the two clusters generated by CLUMPP based on the STRUCTURE v2.2 analysis (K=2).

| **Population** | **Sample size** | **Cluster (inferred gene pool)** | |
| --- | --- | --- | --- |
|  |  | **1** | **2** |
| WL | 29 | 0.9960 | 0.0040 |
| DL1 | 32 | 0.9970 | 0.0030 |
| DL2 | 32 | 0.9960 | 0.0040 |
| DL3 | 32 | 0.9900 | 0.0100 |
| DL4 | 31 | 0.9530 | 0.0470 |
| DL5 | 32 | 0.9770 | 0.0230 |
| BH | 32 | 0.9840 | 0.0160 |
| XB1 | 31 | 0.9970 | 0.0030 |
| XB2 | 30 | 0.9910 | 0.0090 |
| XX | 29 | 0.9970 | 0.0030 |
| BS | 32 | 0.9900 | 0.0100 |
| WD | 32 | 0.9180 | 0.0820 |
| WZH | 30 | 0.9980 | 0.0020 |
| WX | 29 | 0.9980 | 0.0020 |
| WB1 | 32 | 0.9980 | 0.0020 |
| WB2 | 28 | 0.9980 | 0.0020 |
| LYS1 | 32 | 0.9580 | 0.0420 |
| LYS2 | 30 | 0.9940 | 0.0060 |
| HYP1 | 32 | 0.9950 | 0.0050 |
| HYP2 | 32 | 0.9960 | 0.0040 |
| HYP3 | 32 | 0.9960 | 0.0040 |
| HYP4 | 32 | 0.9720 | 0.0280 |
| GD | 32 | 0.9150 | 0.0850 |
| DQ | 31 | 0.9080 | 0.0920 |
| SNJ | 30 | 0.2164 | 0.7836 |
| TB | 30 | 0.0458 | 0.9542 |
| HZZ | 31 | 0.0799 | 0.9201 |
| CQ | 32 | 0.0429 | 0.9571 |
| HL1 | 30 | 0.1406 | 0.8594 |
| HL2 | 30 | 0.1417 | 0.8583 |
| MXS1 | 32 | 0.0020 | 0.9980 |
| MXS2 | 32 | 0.0058 | 0.9942 |
| LHS | 32 | 0.0040 | 0.9960 |
| LD | 32 | 0.0066 | 0.9934 |
| DT | 32 | 0.0060 | 0.9940 |
