# Supplementary material for: Population Genetic Diversity and Structure of a Naturally Isolated Plant Species, Rhodiola dumulosa (Crassulaceae)
Source: PLoS One. 2011 Sep 1;6(9):e24497. doi: 10.1371/journal.pone.0024497 (PMC3164725; doi:10.1371/journal.pone.0024497)
Supplement: Table S2 — Population genetic diversity of R. dumulosa. (n: population size; PPL: proportion of polymorphic loci at the 5% level; Hj: expected heterozygosity or Nei's gene diversity; S.E.: standard error; Var: variance; H: Nei's gene diversity; I: Shannon diversity index; NC: northern China; CNWC: central and northwestern China). (DOC) [file pone.0024497.s002.doc]

Table S2：Population genetic diversity of *R. dumulosa* (n: population size; PPL: proportion of polymorphic loci at the 5％ level; Hj: expected heterozygosity or Nei’s gene diversity; S.E.: standard error; Var: variance; H: Nei’s gene diversity; I: Shannon diversity index; NC: northern China; CNWC: central and northwestern China).

| **Population** | **n** | **PPL** | **Hj** | **S.E.(Hj)** | **Var(Hj)** | **H** | **I** |
| --- | --- | --- | --- | --- | --- | --- | --- |
| DT | 32 | 27.1 | 0.11931 | 0.00947 | 0.000090 | 0.0923 | 0.1490 |
| LD | 32 | 71.6 | 0.18024 | 0.01078 | 0.000116 | 0.1440 | 0.2253 |
| MXS1 | 32 | 21.3 | 0.09690 | 0.00934 | 0.000087 | 0.0742 | 0.1171 |
| MXS2 | 32 | 37.3 | 0.13210 | 0.00939 | 0.000088 | 0.1044 | 0.1707 |
| LHS | 32 | 46.2 | 0.17898 | 0.01063 | 0.000113 | 0.1512 | 0.2399 |
| DQ | 31 | 77.8 | 0.21463 | 0.01157 | 0.000134 | 0.1775 | 0.2698 |
| HL1 | 29 | 38.7 | 0.18113 | 0.01174 | 0.000138 | 0.1428 | 0.2161 |
| HL2 | 29 | 35.6 | 0.16987 | 0.01131 | 0.000128 | 0.1320 | 0.2005 |
| BS | 31 | 39.6 | 0.16786 | 0.01098 | 0.000120 | 0.1332 | 0.2057 |
| WL | 29 | 33.8 | 0.15299 | 0.01171 | 0.000137 | 0.1259 | 0.1896 |
| XB1 | 31 | 29.8 | 0.13086 | 0.01046 | 0.000109 | 0.1049 | 0.1643 |
| XB2 | 29 | 33.8 | 0.13727 | 0.01023 | 0.000105 | 0.1080 | 0.1706 |
| XX | 28 | 32.0 | 0.12531 | 0.00966 | 0.000093 | 0.0993 | 0.1582 |
| BH | 31 | 40.0 | 0.15185 | 0.01046 | 0.000109 | 0.1275 | 0.2006 |
| DL1 | 32 | 31.1 | 0.13715 | 0.01047 | 0.000110 | 0.1069 | 0.1650 |
| DL2 | 31 | 32.9 | 0.15196 | 0.01118 | 0.000125 | 0.1205 | 0.1842 |
| DL3 | 31 | 40.4 | 0.16991 | 0.01106 | 0.000122 | 0.1428 | 0.2218 |
| DL4 | 30 | 69.3 | 0.20997 | 0.01164 | 0.000136 | 0.1850 | 0.2856 |
| DL5 | 31 | 45.3 | 0.18630 | 0.01076 | 0.000116 | 0.1489 | 0.2310 |
| HYP1 | 32 | 35.6 | 0.14415 | 0.01066 | 0.000114 | 0.1155 | 0.1799 |
| HYP2 | 32 | 35.6 | 0.14976 | 0.01104 | 0.000122 | 0.1224 | 0.1892 |
| HYP3 | 32 | 31.6 | 0.14374 | 0.01131 | 0.000128 | 0.1146 | 0.1743 |
| HYP4 | 32 | 37.8 | 0.15605 | 0.01094 | 0.000120 | 0.1265 | 0.1971 |
| LYS1 | 32 | 43.1 | 0.17091 | 0.01095 | 0.000120 | 0.1375 | 0.2139 |
| LYS2 | 30 | 31.6 | 0.13824 | 0.01013 | 0.000103 | 0.1092 | 0.1730 |
| GD | 32 | 38.7 | 0.15521 | 0.01048 | 0.000110 | 0.1225 | 0.1919 |
| WD | 31 | 76.9 | 0.22679 | 0.01079 | 0.000117 | 0.2022 | 0.3174 |
| WZH | 29 | 28.9 | 0.12401 | 0.01015 | 0.000103 | 0.0982 | 0.1530 |
| WX | 29 | 31.1 | 0.13448 | 0.01048 | 0.000110 | 0.1037 | 0.1598 |
| WB1 | 32 | 32.4 | 0.13320 | 0.01039 | 0.000108 | 0.1081 | 0.1688 |
| WB2 | 28 | 28.4 | 0.12508 | 0.00988 | 0.000098 | 0.0969 | 0.1526 |
| TB | 29 | 25.3 | 0.11032 | 0.01002 | 0.000100 | 0.0864 | 0.1340 |
| CQ | 32 | 23.1 | 0.10484 | 0.00958 | 0.000092 | 0.0850 | 0.1341 |
| HZZ | 31 | 27.1 | 0.10936 | 0.00984 | 0.000097 | 0.0853 | 0.1336 |
| SNJ | 30 | 29.3 | 0.12919 | 0.01045 | 0.000109 | 0.1005 | 0.1551 |
| Total | 1089 |  |  |  |  | 0.2473 | 0.3625 |
| NC | 806 |  |  |  |  | 0.1972 | 0.3185 |
| CNWC | 283 |  |  |  |  | 0.2216 | 0.3458 |
